# Improved training tolerance by supplementation with α-Keto acids in untrained young adults: a randomized, double blind, placebo-controlled trial

**DOI:** 10.1186/1550-2783-9-37

**Published:** 2012-08-02

**Authors:** Yuefei Liu, Rupprecht Lange, Juliane Langanky, Thea Hamma, Bingquan Yang, Jürgen M Steinacker

**Affiliations:** 1 Section of Sports and Rehabilitation Medicine, Department of Internal Medicine II, University of Ulm, Ulm, D-89070, Germany; 2 Department of Endocrinology, Zhongda Hospital, Southeast University, Nanjing, 210009, China

## Abstract

**Background:**

Exercise causes a variety of physiological and metabolic changes that can in turn reduce exercise tolerance. One of the potential mechanisms responsible for fatigue is “exercise-induced hyperammonemia”. Previous studies have shown that supplementation with amino acids can increase training tolerance. The α-keto acids are biochemical analogs of amino acids and can be converted to amino acids through transamination, thus reducing the cellular ammonia level. This double blind, placebo-controlled study was designed to investigate the effects of α-keto acid supplementation (KAS) on training tolerance, training effect, and stress-recovery state.

**Methods:**

Thirty-three untrained young male adults underwent four weeks of training (5 sessions/week; 30 minutes running at the individual anaerobic threshold followed by 3 x 3 minute sprints/each session). Throughout the 4 weeks of training and one week of recovery, subjects took α-ketoglutarate (AKG group, 0.2 g/kg/d, n = 9), branched-chain keto acids (BCKA group, 0.2 g/kg/d, n = 12) or isocaloric placebo (control group, n = 12) daily.

**Results:**

The 4^th^ week training volume, maximum power output and muscle torque were higher in the AKG group (175 ± 42 min, 412 ± 49 Watts and 293 ± 58 Newton meters, respectively, P<0.05) and the BCKA group (158 ± 35, 390 ± 29 and 273 ± 47, P<0.05) than in the control group (92 ± 70, 381 ± 67 and 233 ± 43). The general stress and emotional exhaustion as assessed by the rest-stress-questionnaire-sport after the 3^rd^ week of training increased significantly in the control group (P<0.05), but not in the KAS groups.

**Conclusions:**

Under KAS, subjects could bear a higher training volume and reach a higher power output and peak muscle torque, accompanied by a better stress-recovery-state. Thus, KAS improves exercise tolerance and training effects along with a better stress-recovery state. Whether the improved training tolerance by KAS is associated with effects on ammonia homeostasis requires further observation.

## Background

Physical exercise causes diverse physiological challenges, including mechanical strain of the skeletal muscle [1] and molecular responses [2,3], as well as metabolic changes. Among the metabolic changes induced by exercise, blood lactate concentration has been extensively investigated [4,5]. It is well-known that protein breakdown is accelerated with intensive exercise [6]. Under high-intensity exercise, amino acids produced from muscle protein breakdown are partly used to produce energy [7]. It has been shown that the blood level of ammonia increased significantly in rats during resistance exercise and in humans during intense dynamic exercise [8,9]. Several studies have reported that an exercise bout causes a dramatic increase in ammonia concentration along with an increase in inosine-5´-monophosphate (IMP) and the ratio of IMP/AMP (adenosine monophosphate), demonstrating a deamination process from AMP to IMP under high energy turnover [10], which can remain above the baseline level after one hour of recovery [9]. Previous studies have attributed exercise-induced hyperammonemia to fatigue [11,12]. Therefore, an ammonia accumulation caused by exercise is considered a negative factor for exercise tolerance.

The effects of nutritional intervention, especially amino acid supplements, on physical performance have been reported [13]. It is evident that supplementation with specific amino acids, such as glutamate, reduces ammonia concentrations during exercise [14]. However, it is also evident that supplementation with branched-chain amino acids (BCAA) leads to a distinct elevation in arterial ammonia level during 60 min of exercise [15]. Alternatively, α-keto acids that are analogs to amino acids do not donate ammonia during metabolism, but can catch or re-bind ammonia through the ammonia-shuttle or recycle function [16]. For example, α-ketoglutarate (AKG), re-binds ammonia through the action of aminotransferase to form glutamate, and the branched-chain keto acid (BCKA) to form BCAA (the so-called BCKA-BCAA cycle) [16]. As a result, α-keto acids, by exerting biological roles in protein metabolism, may prevent or attenuate the hyperammonemia associated with physical training [17].

Previous studies of nutritional interventions with supplementation of amino acids during physical training have been published. BCAA supplementation was reported to increase endurance capacity in trained individuals [18,19], but this result was not supported by other studies [20,21]. In addition, the combination of the keto analog and amino acid supplementation was reported to attenuate the increase in blood ammonia concentration after an exercise bout [8,22]. However, studies of the effects of α-keto acid supplementation (KAS) seem to be principally limited to pathological conditions such as renal or hepatic disorders, and the effects of KAS alone on physical exercise in healthy subjects remain unknown. Because glutamate/glutamine and BCAA play the prominent roles in protein metabolism and have been extensively investigated [23-25], examining the effects of their keto acid analogs (i.e., AKG and BCKA) on physical training is of scientific interest. We hypothesized that KAS can improve training tolerance under physiological conditions through its biochemical role as an amino acid analog, but without ammonia loading. This study was aimed to investigate the effects of KAS on exercise tolerance, training effect, and stress-recovery state in normal healthy subjects in a double-blind, randomized, placebo-controlled trial.

## Methods

### Subjects

Thirty-six healthy male volunteers were initially enrolled in the study. The health status of the subjects was verified by medical history, physical examination, electrocardiogram, echocardiogram, lung function test with body plethysmogram and routine blood tests (full blood counts, creatine kinase, aspartate transaminase, alanine transaminase, and alkaline phosphatase, as well as electrolytes, glucose, cholesterol and triglycerides) according to the standards of German Society of Sports Medicine. Subjects with obesity, diabetes mellitus, cardiovascular diseases and maple syrup urine disease were excluded. The untrained status of the subjects was considered when the following criteria were all met: physical exercise had not been regular and was less than 2 hours each week during the last three years, and maximum oxygen uptake (VO_2max_) was < 50 ml·min^1^·kg^-1^. After giving informed consent, the subjects were randomized (randomization was generated by the software package SPSS, IBM, USA) into three groups, according to the type of nutritional intervention. Thirty-three subjects completed the study, while three subjects dropped out of the study: one due to his business travel and two who suffered ankle injuries at the beginning of the training due to uneven running tracks. The general information of the subjects is summarized in Table 1. This study was approved by the Ethics Committee of the Medical Faculty of the University of Ulm (Ulm, Germany).

**Table 1:** Basic data of the study subjects (mean ± SD)*

**Group**	**n**	**Age (years)**	**Body mass (kg)**	**Height (cm)**	**BMI (kg/m²)**
Control	12	25.2 ± 6.4	75.9 ± 8.3	179.1 ± 4.9	23.7 ± 2.9
AKG	9	26.7 ± 4.8	81.6 ± 12.7	178.3 ± 8.1	25.6 ± 2.5
BCKA	12	25.1 ± 6.8	78.6 ± 7.5	181.1 ± 4.8	23.9 ± 1.9

*BMI*: Body mass index = body mass (kg) / (body height in meters)²; *AKG*: α-keto glutarate;

*BCKA*: Branched-chain keto acids. * No significant difference between the groups.

### Study design and protocol

**Figure 1:**
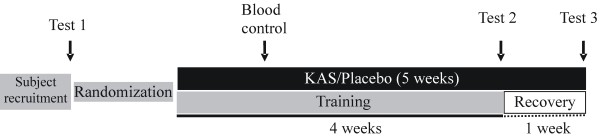
** Study protocol.** After receipt of the informed consent from the subjects, measurements of study parameters were performed at time point 1, 2 and 3. After approximately 1 week of α-keto acid supplement (KAS), blood samples were collected for medical monitoring.

The basic design of this study was a double blind, randomized, placebo-controlled trial. After recruitment, the subjects were randomized into the three groups. Observations were made before and after the training as well as after the recovery. Blood samples were collected 1 week after nutritional supplementation (for medical monitoring). The diet of the subjects was not manipulated but was well documented and analyzed with the software package PRODI (Freiburg, Germany) [26]. The details are described as follows (Figure 1).

### Physical training

The goal of the physical training was to challenge energy metabolism by achieving an “over-reaching” training level [27]. Two parts of physical training were included in each training session: a 30 minute endurance run followed by 3 x 3 minute sprints (maximum speed of the subjects, heart rate ≥ 95% of the maximum on treadmill test). The intensity of the endurance training was set according to the heart rate at the individual anaerobic threshold (IAT) [4] as determined by a treadmill test (see below). The training program was four weeks long with five sessions each week, under supervision. The training was carefully documented and training time was calculated. After the training phase, the subjects underwent a one-week recovery. During the recovery phase, no exercise was enforced except for daily life activities.

### Supplement of α-keto acids

According to the randomization, the subjects took one of the following supplement mixes in granules (~ 2 mm in diameter). The materials for KAS were kindly donated by Evonik Rexim SAS (France) and were packed in small bags containing the individual daily dose for each subject. KAS was orally (with water) given each day over the period from training to the end of the recovery week (5 weeks). The subjects were instructed to take KAS within the time interval two hours before and two hours after training or 16:00 – 20:00 hours on the non-training days. A protocol for KAS, including compliance or complaints, was documented every day by an assistant of the study team.

Mix 1: AKG (0.2 g·kg^-1^·d^-1^), prepared with Na-AKG 144.66 mg·kg^-1^·d^-1^ (correspondingly 127.60 AKG mg·kg^-1^·d^-1^) and Ca-AKG 91.33 mg·kg^-1^·d^-1^ (correspondingly 72.40 mg·kg^-1^·d^-1^ AKG).

Mix 2: BCKA (0.2 g·kg^-1^·d^-1^), composed of three components (α-ketoisocaproate, KIC, 47.4%; α-ketoisovalerate, KIV, 30.0% and α-ketomethylvalerate, KMV, 22.6%), prepared as follows: Na-KIC: 111.47 mg·kg^-1^·d^-1^ (correspondingly KIC 94.80 mg·kg^-1^·d^-1^), Ca-KIV: 69.73 mg·kg^-1^·d^-1^ (correspondingly KIV 60.00 mg·kg^-1^·d^-1^), Ca-KMV: 52.40 mg·kg^-1^·d^-1^ (correspondingly 45.20 mg·kg^-1^·d^-1^).

Mix 3: Placebo of equivalent energy and sodium, as well as calcium salts of the same appearance as AKG and BCKA, composed of 235 mg·kg^-1^·d^-1^ glucose, 41.09 mg·kg^-1^·d^-1^ CaCO_3_, 38.02 mg·kg^-1^·d^-1^ NaHCO_3_.

### Determination of the study parameters

Observations were made at three points (Figure 1): before the training as the baseline (Test 1), after the four weeks of training (Test 2) and at the end of one week of recovery (Test 3). The following parameters were determined.

The weekly training time was calculated for both endurance running and sprint running, according to the training protocol (Figure and Table 2).

**Table 2:** Training data (mean ± SD)

			**Group**	
		**Control**	**a-KG**	**BCKA**
Training time (min/w)				
Endurance training	week 1	144 ± 12	143 ± 13	146 ± 14
	week 2	130 ± 25	127 ± 33	140 ± 15
	week 3	112 ± 48^*^	147 ± 10	127 ± 47
	week 4	74 ± 54^**^	137 ± 30^††^	122 ± 27^††^
sprint running	week 1	44 ± 6	42 ± 4	42 ± 6
	week 2	35 ± 8	37 ± 12	40 ± 6
	week 3	30 ± 17	41 ± 5	34 ± 15
	week 4	19 ± 17^**^	39 ± 12^††^	35 ± 8^†^
VO_2max (ml·min_-1_·kg_-1_)_	before training	45.6 ± 7.3	47.1 ± 6.9	45.4 ± 5.1
	after training	52.3 ± 6.2^‡‡^	52.1 ± 7.2^‡‡^	51.3 ± 5.2^‡‡^
	after recovery	51.9 ± 8.3^‡‡^	52.6 ± 7.1^‡‡^	51.1 ± 5.1^‡‡^
P_max (Watts)_	before training	365 ± 63	380 ± 59	369 ± 34
	after training	377 ± 61	381 ± 56	374 ± 46
	after recovery	381 ± 67	412 ± 49^‡^	390 ± 29^‡^
P_IAT (km/h)_	before training	9.6 ± 1.7	9.8 ± 2.2	9.9 ± 1.5
	after training	10.8 ± 1.7^‡^	10.6 ± 1.7^‡^	10.6 ± 1.6^‡^
	after recovery	10.5 ± 1.7	10.2 ± 2.1	10.4 ± 1.4

*AKG*: α-keto glutarate; *BCKA*: branched-chain keto acids; min/w: training time in minutes each week; *VO*_*2max*_: the maximum oxygen uptake measured on the cycle-ergometry; *P*_*max*_: the maximum power output on the cycle-ergometry; *P*_*IAT*_: the performance at the individual lactate threshold determined by treadmill test; *T*_*max_ISM*_: the maximum muscle torque by isometric measurement; *P*_*max_ISK*_: the maximum muscle performance by isokinetic measurement.

^*^ P<0.05 compared with that of 1^st^ week; ** P<0.01 compared with that of 1^st^ week; ^†^ P<0.05 compared with that of the control group; ^††^ P<0.01 compared with that of the control group; ^‡^ P<0.05 compared with that before training; ^‡‡^ P<0.01 compared with that before training.

VO_2max_: an exercise test was undertaken on a cycle-ergometer (Lode Excalibur, Lode Company, Netherlands) with the rapid ramp protocol (rapid linear increment at 40 Watts·min^-1^) until the subject was exhausted. The oxygen uptake was measured breath-by-breath using a Metamax 3B (Cortex Company, Germany). Maximum power output (P_max_) and VO_2max_ were derived from this test.

Running performance at the IAT [4] was determined by a standard treadmill test (incline 1.5%, beginning at 6 km·h^-1^, increment 2 km·h^-1^ every 3 min) until the subject was exhausted. Performance at the IAT (P_IAT_) was calculated from the relationship between power output and changes in blood lactate concentration [4].

The isometric maximum torque (T_max_ISM_) and isokinetic maximum performance (P_max_ISK_) of the quadriceps femoris of the dominant leg were determined using an Isokinetic BIODEX Dynamometer (Biodex Medical Systems, USA); the maximum value was taken from three attempts. T_max_ISM_ was tested with the knee extension at position 90°, and P_max_ISK_ with the start position at 90° and 60°·s^-1^ rotation, according to the manufacturer’s instructions.

### Stress and recovery state

To monitor status and changes in stress and recovery of the subjects during the study period, a recovery-stress questionnaire (RESTQ-Sport) was used. The RESTQ-Sport was specifically developed to measure the frequency of current stress and recovery-associated activities, and the German version of the RESTQ-Sport consists of 76 items (19 scales with four items each). A Likert-type scale was used, with values ranging from 0 (never) to 5 (always) (for the details please refer to [28]). The questionnaires were completed weekly by the subjects.

### Data analysis and statistics

All data are expressed as the mean ± SD; a P<0.05 was considered as statistically significant, using an analysis of variance with a post-hoc Scheffé test.

## Results

During the study, no complaints or complications related to KAS were reported. No pathological changes or differences among the groups were found in the clinical laboratory parameters. The subjects’ compliance with taking the nutritional supplement was satisfactory (98.3%).

The diet was comparable among the different groups and did not change throughout the study period (total caloric intake: 2509 ± 115 kcal·d^-1^, of which carbohydrates composed 49.2%; fat 30.3%; protein 17.1% and alcohol 3.4%).

Metabolic parameters, including BMI, body fat percentage (15.9 ± 0.7%) measured by infrared spectrometer, blood concentration of glucose (4.7 ± 0.1 mmol·l^-1^), cholesterol (4.3 ± 0.1 mmol·l^-1^), triglyceride (1.6 ± 0.1 mmol·l^-1^) and C-reactive protein (0.8 ± 0.1 mg·l^-1^), were similar among the different groups.

### Training

**Figure 2:**
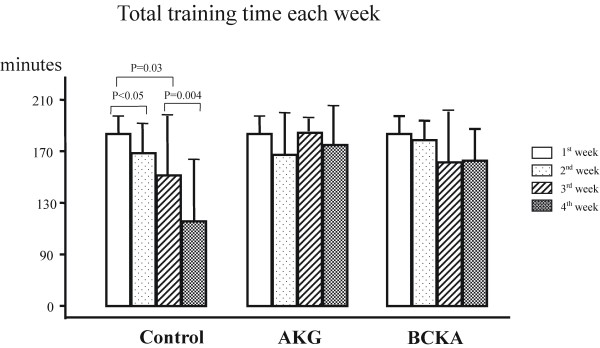
** The total training time (minutes) each week in mean ± SD (endurance training time + sprint running time).** AKG: α-keto glutarate; BCKA: branched-chain keto acids.

**Figure 3:**
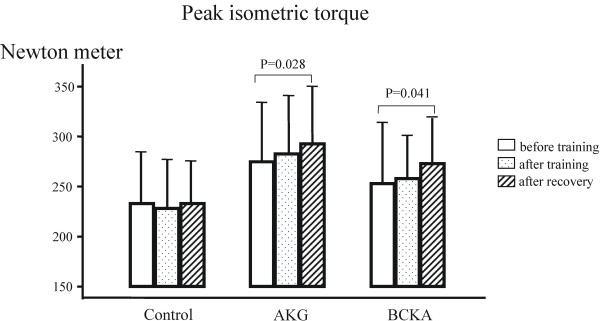
The peak maximal isometric torque (Newton meter) in mean ± SD. AKG: α-keto glutarate; BCKA: branched-chain keto acids.

**Figure 4:**
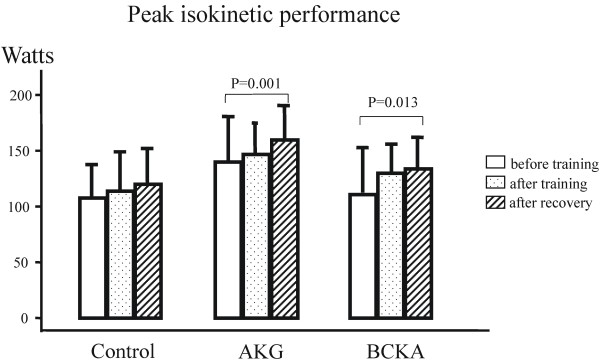
** The peak isokinetic performance (Watts) in mean ± SD.** AKG: α-keto glutarate; BCKA: branched-chain keto acids.

The training data are summarized in Table 2 and Figures 2, 3 and 4. During the first two weeks of training, the total training time, training times for endurance and for sprint running did not differ significantly among the groups. However, from the third week onwards all training times decreased in the control group (P<0.05), while they remained essentially unchanged in the AKG group and the BCKA group (Figure 2). Thus, the training volume in the second half of the training phase was clearly lower in the control group than in the AKG and the BCKA groups (P<0.05).

The VO_2max_ increased significantly after training and during recovery in all three groups (P<0.01), and there was no significant difference among the three groups at each test time point. The P_max_ increased in the groups supplemented with KAS after the recovery period compared with that before training (P<0.05), while the increase in P_max_ in the control group was less and was not statistically significant. The endurance capacity assessed by P_IAT_ was increased at the end of training in all three groups, but no statistically significant difference was observed among the groups.

The muscle function tests showed that the isometric maximum torque was different at the baseline level among the groups, but the difference was not statistically significant (P = 0.27). The torque did not change in the control group after training and recovery, but it increased significantly after the recovery week (P<0.05) in the AKG and BCKA groups (Figure 3). Similar results were observed in muscle performance as assessed by the isokinetic measurement (Figure 4). The baseline level of muscle performance was different among the groups, but the difference was not statistically significant (P = 0.144).

### Stress-recovery state

**Figure 5:**
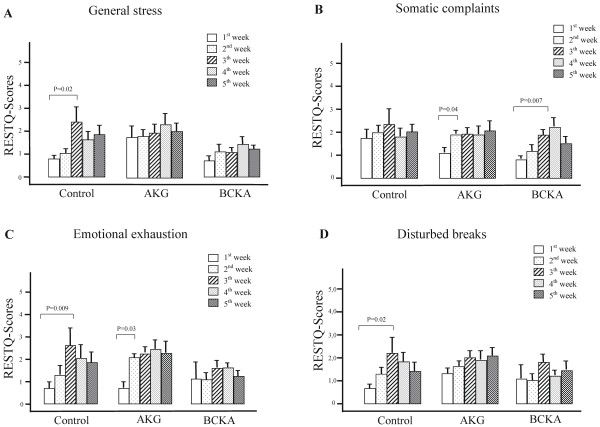
** The weekly data from the recovery-stress questionnaire (RESTQ-scores) for general stress (A), somatic complaints (B), emotional exhaustion (C) and disturbed breaks (D).** AKG: α-keto glutarate; BCKA: branched-chain keto acids. RESTQ-Scores 0: never; RESTQ-Scores 5: always.

In the RESTQ-Sport analysis, the general stress was markedly increased in the control group during the third week of the training (P<0.05) (Figure 5a), and it did not change in BCKA group (NS). In the AKG group, the general stress was higher at baseline than in the other groups, but it did not change significantly during the study period (NS).

For the somatic complaints (Figure 5b), the baseline RESTQ-scores in the control group were higher (but not statistically significant) than in the other groups. These values were essentially unchanged during the entire observation period in the control group, while increasing in the AKG group (P<0.05) and in the BCKA group (P<0.01) during the training phase.

Emotional exhaustion assessed by RESTQ-scores increased and reached the highest level during the third week of the training in the control group (P<0.01) (Figure 5c) but did not change significantly in the BCKA group (NS). In the AKG group, it was elevated during the last three weeks of the training as well as after the recovery compared with the before-training scores (P<0.05).

The RESTQ-scores for the disturbed breaks increased from the 1^st^ to the 3^rd^ week of training (P<0.05), and then decreased gradually in the control group (Figure 5d). There was no change in the AKG or the BCKA group during the observation period, although there were more disturbed breaks in the AKG group than in the BCKA group.

## Discussion

Physical exercise causes a variety of physiological changes that in turn impact exercise tolerance. An accumulation of metabolites such as ammonia produced by deamination from AMP to IMP and by protein metabolism during exercise may play an important role in this regard. Any modification to metabolites may affect exercise tolerance. Previous studies have shown that supplementation with amino acids can lead to changes in energy metabolites and physical performance [18,29-32].

Biochemically, α-keto acids are endogenous intermediate metabolites, analogs to amino acids and may affect the cellular and blood level of ammonia [33-36]. Therefore, it is likely that supplementation with α-keto acids has an impact on physical training. We have therefore hypothesized that supplementation with α-keto acids improves exercise tolerance and training effects. In this study, we found that by supplementing the subjects with KAS, their training volume, maximum power output and maximum muscle torque, as well as their performance, were all significantly increased, which was associated with a better recovery-stress state. Therefore, KAS can indeed improve training tolerance.

### KAS effects on physical training

A number of studies of nutritional intervention during physical training have been published. A recent study reported that acute supplementation of cyclists with keto analogs and amino acids during exercise attenuated exercise-induced hyperammonemia [22]. However, the effects of KAS alone during prolonged physical training have not been reported. In the present study, we have adopted the double blind, randomized and placebo-controlled trial design, so that the subjective component affecting exercise tolerance could be precluded from the effects of KAS. To provoke the metabolic challenge, a cohort of untrained subjects was recruited and a very strenuous training program was undertaken to achieve an “over-reaching” status. The training was highly demanding; the subjects in the control group could not maintain their assigned training volume during the second half of the program (Table 2, Figure 2,3 and 4). The training data also showed a typical training effect at the stage of over-reaching; i.e., a significant improvement in maximum power output after recovery but only slightly in aerobic exercise capacity, as previously reported [37]. The subjects underwent an endurance-training bout first so that the energy reserve was exhausted, and the subsequent sprint running would then draw energy partly from protein metabolism. This training strategy constitutes a valid metabolic challenge [6]. To exclude the influence of components other than α-keto acids, the intake of energy and minerals was carefully matched in the placebo preparation. There were no side effects or difficulties in compliance, suggesting that the supplementation was safe.

Despite the hard training, over-training did not occur because there were no clinical complaints and no decrease in the maximum performance and maximum blood lactate concentration (10.7 ± 2.4 mM). The training, however, improved VO_2max_ (average 14%, P<0.01) in all three groups (Table 2). This result is in accord with those of other studies [38]. The training effect on VO_2max_ was comparable among the three groups, although the training volume was quite different at the second half of the training phase. This finding may be explained by the fact that the oxygen delivery determined principally by the cardiorespiratory system is the primary limiting factor for VO_2max_[39]. The maximum power output did not change in the control group after the training phase and recovery (NS). There was a similar increase in maximum power output in both study groups after the training and more so after recovery, indicating a “super-compensation” effect from training (Table 2). These results are in good accord with those of previous studies [40], and suggest a significant training effect in both groups supplemented with KAS. Similarly, the muscle function, both maximum torque on isometric measurement and maximum performance on isokinetic measurement, increased significantly after recovery in both groups supplemented with KAS. The maximum muscle torque was higher in the AKG group than in the BCKA group (Figure 3), mainly due to the different baseline levels but not changes in training (NS). In the present study, the endurance capacity (P_LAT_ in Table 2) was improved in all three groups with no significant difference among the groups, which could be attributed to the concurrent training program executed with combined training components [41].

It is also interesting to observe the relative changes in VO_2max_ and P_max._. There was a similar increase in VO_2max_ in all three groups, but the P_max_ was much higher in the two groups with KAS than in the control group, suggesting that there was either a higher work efficiency or a higher quotient of anaerobic energy metabolism associated with KAS. Because the maximum blood lactate concentration was comparable among the groups (data not shown), the higher relation of P_max_ to VO_2max_ for both groups with KAS can be considered as reflecting improved work efficiency.

VO_2max_ was determined on a cycle-ergometer instead of using a treadmill test since this method was established in our laboratory and a rapid linear increment of the workload was better to achieve. Determination of VO_2max_ on a cycle-ergometer is well established and widespread in the routine practice of sports medicine. Yet it remains unclear whether a lack of statistically significant difference in this parameter among the groups could be attributed to the known fact that VO_2max_ determined on a cycle-ergometer is generally lower than that determined on a treadmill [42,43].

In summary, the training program performed in this study produced distinct training effects in the control group. However, KAS supplementation was associated with additional improvements in P_max_ and maximum muscular torque and performance. Together with the data from training volume, it can be concluded that KAS improves training tolerance and has beneficial effects on physical training.

### KAS effects on stress-recovery state

The state of stress-recovery during and after a training phase can be assessed using the questionnaire RESTQ-sport [28]. In general, the profiles of the RESTQ scores were quite different among the three groups (Figure 5A-D). The term general stress reached its highest level in the control group after the third training week (Figure 5A). Emotional exhaustion (Figure 5C) and a slight increase in somatic complaints (Figure 5B) followed the same pattern but with distinct disturbed breaks as a sign of poor recovery (Figure 5D). A decrease in the general stress parameters at the end of the 4^th^ training week and after recovery was associated with a reduction in training volume (Figure 2). This finding is in agreement with those of Kellmann and Gunther, who concluded that the general stress and somatic complaints were correlated with the duration of intense training [28]. In contrast with the results for the control group, the RESTQ scores for the terms general stress (Figure 5A) and emotional exhaustion (Figure 5C) in the BCKA group did not change significantly and remained at a lower level, but the somatic complaints increased during the training period (Figure 5B). These data suggest that BCKA supplements can relieve general stress and emotional exhaustion and better preserve the recovery after high-level exercise. With the AKG supplement, the RESTQ profile was comparable to that of the control group, although the training volume was higher in the 3^rd^ and 4^th^ training weeks. Considering the relationship between the amount of training and RESTQ scores in general stress and somatic complaints reported by Kellmann and Gunther [28], our data suggest that supplementation with AKG helps maintain the level of general stress, somatic complaints and emotional exhaustion during high-intensity training.

To the best of our knowledge, there are no previous studies investigating the effects of KAS supplementation on physical training. However, two relevant studies have been reported [8,22]. In a study of adult rats, De Almeida et al. have shown that exercise increased ammonia levels twofold with respect to the control and significantly increased blood urea levels (17%). Those authors also report that acute supplementation with keto acid-associated amino acids (KAAA) clearly reduced exercise-induced hyperammonemia [8]. This result was supported by a study of male cyclists, where it was observed that short-term supplementation with KAAA blunted exercise-induced hyperammonemia [22]. However, in these two acute studies, the effect of KAAA on exercise tolerance was not investigated. Thus, whether the inhibition of exercise-induced hyperammonemia by supplementation with KAAA leads to an improvement in training tolerance remains unclear. Although the underlying mechanism of the effects of the supplementation of α-keto acids on physical exercise remains unclear, we have shown the beneficial impact of the supplementation with KAS on physical training in untrained individuals. Further studies are needed to clarify whether KAS supplementation affects amino acid homeostasis and ammonia metabolism during and after physical exercise.

## Conclusions

Physical exercise is of great significance to public health. However, to maintain physical activity is by no means simple, and exercise adherence is affected by a variety of factors. Finding ways to modify inhibitory factors such as exercise-induced hyperammonemia is of great scientific and clinical interest. This study has shown that nutritional supplementation with α-keto acids in healthy, untrained subjects significantly improved exercise tolerance, training effects, and stress-recovery state. Therefore, observations to further verify the potential benefits of α-keto acid supplements in subjects during active training will be of scientific and clinical value.

## Competing interests

The authors declare that they have no competing interests.

## Authors’ contributions

YL designed the study, conducted the investigations and analyzed the data; RL and JL recruited the subjects and guided the physical training and nutritional supplementation; TH and BY assessed laboratory variables and collected data; JMS coordinated the study. All authors have read and approved the final manuscript.
